# A Worked Example of Qualitative Descriptive Design: A Step‐by‐Step Guide for Novice and Early Career Researchers

**DOI:** 10.1111/jan.16481

**Published:** 2024-10-09

**Authors:** Princess Villamin, Violeta Lopez, Deependra Kaji Thapa, Michelle Cleary

**Affiliations:** ^1^ School of Nursing, Midwifery and Social Sciences CQUniversity Australia; ^2^ Department of Epidemiology and Biostatistics, School of Public Health ‐ Bloomington Indiana University Bloomington 47405 Indiana USA

**Keywords:** early career researcher, novice researcher, nursing, postgraduate researcher, qualitative descriptive, qualitative method, qualitative research design, research method

## Abstract

**Aim:**

To provide a worked example informed by relevant literature and related studies that novice and early career researchers may use to reflect on, prepare and conduct a thoughtful and rigorous qualitative descriptive study.

**Design:**

Methodological discussion of qualitative descriptive design.

**Methods:**

Seminal work and recent related literature were reviewed to situate the discussion and identify the concepts and steps to conduct a qualitative descriptive study.

**Results:**

Qualitative descriptive design is widely used in nursing and health science research. This design offers flexible use of qualitative methods, which presents a double‐edged sword, posing challenges in preparing a well‐developed study and achieving methodological rigour. The design often borrows methods from other qualitative traditions, which may need to be clarified for novice and early career researchers, wherein studies may be conducted using a mix and match of methods without giving justice to the heart of qualitative descriptive design. In this paper, we present a step‐by‐step guide, using a worked example, to demonstrate how to conduct a qualitative descriptive study.

**Conclusion:**

Qualitative descriptive design may be confusing due to its flexibility, which may limit the scope of research and subsequently, the quality and impact of the findings. With the appropriate application of research methods producing high‐quality and relevant findings, qualitative descriptive design is a valuable qualitative method in its own right.

**Implications for the Profession:**

Novice and early career researchers may increase the impact of their findings through rigorously conducting their studies. Clarifying steps for thoughtful execution may inform novice and early career researchers, allowing for a rigorous application of the method, which, in turn, may contribute to impactful findings.

**Impact:**

A clear presentation of steps, supported by a worked example and related studies, may support novice and early career researchers in conducting a qualitative descriptive study with methodological rigour.

**Reporting Method:**

Not applicable.

**Patient or Public Contribution:**

No patient or public contribution.

## 1 Introduction

One of the most commonly used yet misunderstood research designs is qualitative descriptive design or qualitative description (Turale 2020). Although this design is prevalent in nursing and midwifery studies and other health sciences (Bradshaw, Atkinson, and Doody 2017; Doyle et al. 2020), a systematic review of qualitative descriptive studies identified inconsistencies in how the studies were conducted, including variability in when and how methods were adopted and applied from other qualitative traditions (Kim, Sefcik, and Bradway 2017). This article aims to clarify each step in the design, identify key conflicts and sources of confusion and present the process of conducting a qualitative descriptive study, providing tips to support novice and early career researchers in conducting a qualitative descriptive study with methodological rigour. Qualitative descriptive design borrows methods from other qualitative traditions offering much flexibility in its methods. As Sandelowski (2010) reports, ‘In the actual world of research practice, methods bleed into each other’ (p. 81). Nonetheless, the flexibility and variability of qualitative description may confuse those unfamiliar with the design, questioning themselves to identify what qualitative approach they have used (Kim, Sefcik, and Bradway 2017; Neergaard et al. 2009; Turale 2020).

Novice and early career researchers may need explicit steps to guide them through extant literature and clarify current conflicts in qualitative inquiry that may affect how they conduct qualitative descriptive studies. There is a need to clarify the steps involved in a qualitative descriptive study to avoid researchers either claiming to have used other well‐known qualitative methodologies when they have not (to be more scholarly acceptable) or to avoid qualitative descriptive design being attributed to a poorly constructed research study that does not meet classic qualitative traditions (Sandelowski 2010; Turale 2020). Drawing from Sandelowski's (2000) seminal work on qualitative description and informed by recent literature, we aim to clarify the steps used to conduct a qualitative descriptive study and provide a framework of reference to novice and early career researchers. In this article, we discuss and present the steps for conducting a qualitative descriptive study, providing a worked example (experiences and motivations of migrant nurses) to support novice and early career researchers in conducting a qualitative descriptive study with methodological rigour.

## 2 Background

Qualitative descriptive design or qualitative description is a widely used approach in nursing and health sciences (Bradshaw, Atkinson, and Doody 2017). This design aims to generate data on experiences or events from the participants' perspectives, allowing researchers to stay ‘data‐near’ and gain a comprehensive understanding of the phenomena without necessarily focusing on the study of lived experiences or culture or developing theory (Bradshaw, Atkinson, and Doody 2017; Doyle et al. 2020; Sandelowski 2010). Staying close to the participants' rich descriptions makes this design useful in several ways, especially when first‐hand experiences are sought (Neergaard et al. 2009).

While other qualitative approaches, like ethnography, phenomenology or grounded theory, have descriptive components, they also involve high‐level interpretation intending to explain phenomena (Lambert and Lambert 2012; Roudsari 2019). Notwithstanding this, qualitative descriptive studies require a level of interpretation, where the extent of interpretation is dependent on answering the research question consistent with how participants have described the event without moving too far from the data (Bradshaw, Atkinson, and Doody 2017; Doyle et al. 2020; Neergaard et al. 2009; Sandelowski 2010). Findings from a well‐conducted qualitative descriptive study should provide a thorough summary of an event that fundamentally sets the record straight on what the events mean for the participants (Sandelowski 2000).

Thoughtful application of a qualitative descriptive design in research may yield findings that may contribute to improving practice and patient outcomes (Sullivan‐Bolyai, Bova, and Harper 2005; Turale 2020). Meaningful descriptive responses to the ‘what’, ‘why’ and ‘how’ of participants' experiences or perceptions may inform policy development and evidence implementation (Chafe 2017; Colorafi and Evans 2016; Neergaard et al. 2009). By staying true to participant descriptions, qualitative descriptive studies may provide a foundation for larger‐scale studies and the development of questionnaires and intervention studies (Bradshaw, Atkinson, and Doody 2017; Doyle et al. 2020; Neergaard et al. 2009).

## 3 Data Sources

Seminal work on qualitative descriptive design (Sandelowski 2000, 2010), along with recent literature that provides an overview or describes the method, were reviewed (Bradshaw, Atkinson, and Doody 2017; Colorafi and Evans 2016; Doyle et al. 2020; Kim, Sefcik, and Bradway 2017; Sullivan‐Bolyai and Bova 2021). To clarify each step of the method and provide relevant application examples in actual studies, a database search using CINAHL and PubMed was performed (October 2023). However, a systematic review across databases was not conducted. Search terms included ‘qualitative descriptive’, ‘qualitative description’ and ‘nurs*’. No limit was set on the publication date to avoid excluding relevant studies. Bibliographic mining of articles within selected studies was also done to explore further relevant information.

## 4 Discussion

### 4.1 Considerations Before and During the Study

Philosophical paradigms, the anticipated role of theory and ethics must be considered before conceptualising the research. While a thorough explanation of these is beyond the scope of this article, this section presents an overview of philosophical paradigms, the role of theory and ethics as applicable to qualitative descriptive studies.

#### 4.1.1 Philosophical Considerations

Qualitative descriptive design tends to align with the principles of naturalistic inquiry, enabling researchers to study phenomena, events or experiences in their natural state to the extent possible within the context of the study (Lambert and Lambert 2012; Sandelowski 2000). Findings from qualitative descriptive studies contain both a straightforward and comprehensive description of events resulting from the interaction between participants and researcher, and the researcher's understanding of the meaning individual participants ascribe to events (Bradshaw, Atkinson, and Doody 2017; Cutler, Halcomb, and Sim 2021; Sandelowski 2000). This is consistent with a subjectivist epistemology and relativist ontology (Bradshaw, Atkinson, and Doody 2017; Roudsari 2019).

Qualitative description may also be aligned with pragmatism in which the researcher has flexibility throughout the research process (Doyle et al. 2020; Neergaard et al. 2009). The flexible approach of this design allows some elements of other qualitative approaches to be employed, such as methods used in grounded theory, phenomenology, ethnography or narrative inquiry (Sandelowski 2000). With proper justification of choices throughout the study, pragmatism allows researchers to choose methods that answer the research questions while staying close to the participants' descriptions (Doyle et al. 2020; Neergaard et al. 2009).

#### 4.1.2 Theoretical Considerations

The alignment of qualitative descriptive studies and naturalistic inquiry has misled some into viewing qualitative descriptive design as atheoretical (Sandelowski 2010). This may stem from the belief that being guided by a theory may impose preconceptions and alter the naturalistic understanding of the phenomena (Nguyen et al. 2022). Then again, no study can be entered ‘conceptually naked’ without the influence of any theory (Sandelowski 2010, p.79), and some scholars believe that the absence of theoretical frameworks may result in repetitive and purely descriptive information that may have little to contribute to knowledge formation (Collins and Stockton 2018; Nguyen et al. 2022). Whether theory is viewed as enhancing or stifling to research, generally, theory in qualitative research is present in three forms: (1) as a guide to selecting research paradigms and methods, (2) as a result of research or data collection, or (3) as a framework guiding the study (Collins and Stockton 2018; Pope and Mays 2020). Instead of declaring the absence of theory in their studies, researchers should be clear about the role of theory in their inquiry (Nguyen et al. 2022; Sandelowski 2010).

In qualitative descriptive studies, theoretical frameworks may guide the researcher, such as in developing the interview guide, guiding data collection and analysis or writing the discussion (Kim, Sefcik, and Bradway 2017; Sullivan‐Bolyai and Bova 2021). Theoretical frameworks may serve as maps, directing attention to a particular phenomenon and leading researchers to paths that may otherwise be missed (Garvey and Jones 2021; Sandelowski 1993a). For instance, to study the cultural challenges among migrant nurses in the Kingdom of Saudi Arabia, Alosaimi and Ahmad (2016) used Leininger's Culture Care Theory (1996) to explore aspects of culture relevant to their study. This informed their research and enabled them to explore communication patterns between non‐Arabic speaking nurses and Muslim patients and identify barriers to providing care, such as religious and cultural differences (Alosaimi and Ahmad 2016). In our current worked example of a study on retention of migrant nurses, we used the self‐determination theory (SDT) to frame part of our research question, inform our interview guide and support our discussion. Our findings were descriptive to stay true to participants' experiences, and our discussion section was synthesised in relation to SDT and the broader literature. Another study, similarly, by Kelleher, FitzGerald, and Hegarty (2016), explored factors influencing nursing and midwifery students' intentions to study overseas and used the Theory of Planned Behaviour (Ajzen 1985) to guide their open‐ended questionnaire and data analysis. Whilst their findings remained descriptive, they were categorised in accordance with the Theory of Planned Behaviour (their chosen framework) to describe students' intentions and perceptions about studying overseas.

Despite the role of theory in qualitative inquiry, scholars argue that there is a risk of overreliance on theory, which may stifle the data or lead to biased findings to make the framework fit (Garvey and Jones 2021; Morse 1992). The merit of qualitative description is that it facilitates flexibility around the use of theory and commitment to a theory when developing and conducting the study (Kim, Sefcik, and Bradway 2017; Sandelowski 2010). To stay true to qualitative description, researchers do not need to remain committed to a theory or framework if the course of the investigation leads to a path inconsistent with the theory or framework (Colorafi and Evans 2016; Sandelowski 2010). This is not to say that researchers cannot change frameworks during the research or that frameworks can be constantly changed throughout the study; instead, if the findings do not fit the framework, researchers may abandon the framework after clearly stating the reasons, or they may revise the framework depending on the study findings (Sullivan‐Bolyai and Bova 2021). For example, in exploring how migrant nurses in Australia constructed their identities, Zhong et al. (2023) suspended their use of Gidden's theoretical framework to complete a descriptive study of the migrant nurses' transition and adaptation experiences. This allowed them to describe migrant nurses' perceived differences in nursing, adaptation challenges and self‐evolution.

#### 4.1.3 Ethical Considerations

All research requires review by an ethics committee to protect participants, manage risks and uphold ethical standards (Gelling 2016; Polit and Beck 2022; World Health Organisation 2023). This section discusses some ethical considerations in qualitative descriptive studies.

Specific considerations for confidentiality, consent, and power imbalance exist in any qualitative research (Moriña 2020; Dyar 2021). Confidentiality ensures that participants' data will not be disclosed to anyone apart from the researchers (Saunders, Kitzinger, and Kitzinger 2015). This differs from anonymity, where there is ultimately no way for anyone, including the researchers, to link the data to participants (Dyar 2021). As qualitative research requires participant and researcher interaction, participant protection is primarily achieved through maintaining confidentiality by deidentifying data so that collected data is not linked to participants (Cypress 2019; Dyar 2021; Saunders, Kitzinger, and Kitzinger 2015).

While qualitative descriptive studies require thick descriptions of participants, settings and context to aid in transferability, there is a risk of violating confidentiality when these descriptions are recognised by others (Moriña 2020). Therefore, the descriptions of participants and the research setting should be broad, especially when the sample is small and specific. For example, in our current research, when describing the experiences of migrant nurses in a regional hospital, the location would be described as a regional area and migrant nurses would not be identified according to their source country or clinical area to avoid describing participant features or identifying information that risks participant disclosure.

Consent is a fundamental part of ethics and requires voluntary participation based on adequate information and understanding of the study's nature, purpose, methods, expectations from participants, and the implications and potential risks that may arise from participation (National Statement on Ethical Conduct in Human Research 2023; Polit and Beck 2022). This information is often relayed in participant information sheets in a manner suitable to the participant's level of understanding, containing thorough information about the purpose and expectations of the study, their freedom to participate, and their right to withdraw without any consequence (Doody and Noonan 2016; National Statement on Ethical Conduct in Human Research 2023). Participants are viewed as individuals who can make decisions without coercion (Doody and Noonan 2016; Moriña 2020). Researchers communicating information and obtaining verbal or written consent enable participants to ask questions to fully understand their participation (National Statement on Ethical Conduct in Human Research 2023). Some qualitative research may require multiple contacts with participants; thus, whether one consent is enough raises an ethical concern (Dyar 2021; Moriña 2020). Researchers should be mindful that consent may need to be continuously obtained (Cypress 2019). This is sometimes referred to as process consent, rolling informed consent and provisional consent (Flewitt 2005; Piper and Simons 2005; Ramcharan and Cutleffe 2001).

Researchers are also responsible for recruiting fairly, not burdening the participants, and considering the possible power relationship between them and participants (Doody and Noonan 2016; National Statement on Ethical Conduct in Human Research 2023). For example, a researcher interviewing a colleague, a student or their manager may influence responses due to the relationship, including the power differential. To overcome this, researchers should consider involving an interviewer with no prior relationship with the participants. Additionally, researchers should schedule the focus group session or interview at a time suitable for the participants, build trust and rapport through prolonged engagement and discussion, have repeated discussions about the interpretation of findings and openly acknowledge participants' responses (Allmark et al. 2009; Harrowing et al. 2010). Researchers should also reflect on their role as interviewers, the potential for bias during interviews, and be cautious of the verbal and non‐verbal language used throughout the interview (Kostovicova and Knott 2022).

Ethical considerations do not end after obtaining ethical approval. Instead, researchers should maintain ethical practices throughout the research process, proactively identifying potential ethical issues and applying appropriate measures to mitigate these during the course of the study. For further guidance, Carpenter (2011) presents a checklist for assessing the ethical aspects of a qualitative study, while Gelling (1999) enumerates the seven ethical principles that should be maintained throughout any research process.

### 4.2 Steps to Conducting a Qualitative Descriptive Study

This section presents the steps of conducting a qualitative descriptive study after the researcher has formulated a research question and identified that a qualitative descriptive design is most suited to answering it. In the succeeding sections, we provide a worked example of a qualitative descriptive study, supported by a question that novice and early career researchers may ask themselves when planning and conducting the study. We will also acknowledge conflicts and tensions in the current literature of qualitative inquiry, which we will discuss as applicable in the relevant step.

1. Identify the research aim and specify the research question.

Where is the gap in knowledge? What do I want to know?

Conducting any study begins with a well‐formulated research question (Ratan, Anand, and Ratan 2019). Explaining this step is beyond the scope of this article, although multiple papers elaborate on this (e.g., see Agee 2009; Cleary, Hunt, and Horsfall 2011; Doody and Bailey 2016; Mantzoukas 2008; Ratan, Anand, and Ratan 2019). Deciding on the topic and question before the approach is crucial, as incoherence may occur throughout the research if done the other way around (Cleary, Hunt, and Horsfall 2011). In our current study, our research aim was to explore migrant nurses' motivations for migration and experiences during migration and transition to understand factors that lead to workplace retention.

2 Ensure the research aim/question/objective is congruent with a qualitative descriptive design.

Will qualitative descriptive design help me answer my research question?

The flexibility of qualitative description presents a double‐edged sword, with the eclectic combination of methods risking blurring boundaries with other traditional qualitative methods. The mixing and matching of methods conflict with traditional qualitative methods, which are built on a prescribed set of techniques and procedures (Colorafi and Evans 2016). Qualitative description may utilise techniques associated with phenomenology, ethnography or grounded theory; however, this design's outcome does not result in a new theory, produce any phenomenological rendering of the studied phenomenon, or result in a detailed understanding of societal functions within one culture (Sandelowski 2000). If a researcher's aim for a study is to create theory, explore lived experiences or explore cultural phenomena in one's natural setting, then a qualitative descriptive design is inappropriate.

A clear delineation and justification of methods throughout the study helps researchers stay true to qualitative description within its boundaries, which, in turn, adds to the study's credibility (Bradshaw, Atkinson, and Doody 2017; Doyle et al. 2020) instead of appearing as a ‘half‐baked’ grounded theory, ethnographic or phenomenological study. Researchers must justify the choice of the method to contribute to research transparency and enhance rigour (Kim, Sefcik, and Bradway 2017). Doyle et al. (2020) discuss the justification for using a qualitative descriptive design, such as to provide straightforward events or accounts where little is known or to validate findings in a mixed‐methods study. We chose this design to meet our aim of exploring migrant nurses' motivations and experiences during migration and transition and understanding how these experiences contribute to workplace retention. This design allowed us to maintain a straightforward depiction of events and directly identify areas that may inform quality improvement or policy change regarding the migrant nursing workforce (Chafe 2017).

3 Identify the sampling method(s).

Who will accurately describe the event, experience or phenomenon being studied to answer the research question? How will I access them?

Sampling in qualitative research aims to acquire participants or cases to provide detailed and rich data to answer the research question (Polit and Beck 2022). As sampling aims to recruit enough participants to gain a comprehensive understanding and allow a detailed exploration of the phenomenon being studied, researchers may use more than one approach to sampling (Farrugia 2019; Gill 2020; Hennink and Kaiser 2022). Qualitative descriptive studies can use any sampling technique (or a combination of them), wherein the most commonly used techniques are purposive sampling or variations of purposive sampling, including maximum variation and snowball sampling (Doyle et al. 2020; Kim, Sefcik, and Bradway 2017).

Purposive sampling utilises the researcher's judgement to identify and purposefully select participants who may provide comprehensive information on the topic being studied (Farrugia 2019; Gill 2020). Maximum variation sampling, a variant of purposive sampling, involves identifying critical dimensions of the variations, setting the kinds of variation to be maximised (e.g., age, culture, educational level), and selecting cases that vary from each other as much as possible to include a variety of participant characteristics, experiences and perceptions (Kim, Sefcik, and Bradway 2017; Patton 2015; Sandelowski 1995a). On the other hand, snowball sampling involves identifying participants through suggestions or recommendations of initial participants and is beneficial for locating information‐rich participants who could otherwise be difficult to reach or may not initially identify themselves with the phenomenon being studied (Farrugia 2019; Patton 2015). While convenience sampling may also be used, this may be the least recommended because not all participants conveniently identified may provide crucial and relevant information (Kim, Sefcik, and Bradway 2017; Patton 2002).

Since our research aim was to identify migrant nurses' motivations and experiences during migration and transition, our sample had to include migrant nurses. Thus, we employed purposive sampling to access participants who fit our inclusion criteria (i.e., migrant nurses employed in the study setting who migrated within our defined timeframe). Additionally, we wanted to reach as many participants as possible in our limited study setting; hence, snowball sampling was utilised for this purpose, allowing us to gain more participants. Our limited setting, however, meant that we could not employ maximum variation sampling. Nonetheless, the use of both purposive and snowball sampling was sufficient for our study.

*Caveat: Ensure sampling techniques do not place rigid boundaries for participant inclusion*.

Drawing clear boundaries for participant inclusion is essential; however, researchers should be mindful that setting rigid criteria may inadvertently result in missed perspectives (Morse 2010). For instance, a study describing the transition and employment experiences of migrant nurses may report uncomplicated and smooth transitions into their respective areas. Then again, a closer look at participant characteristics may show that all migrant nurses are currently employed in areas that align with their previous clinical experience. Migrant nurses previously employed in specialty areas may have feelings of professional loss when assigned to a different clinical area, which may elicit a negative workplace experience (Ng Chok et al. 2018; Villamin et al. 2023). Hence, researchers may ask questions such as: What about the transition and employment experiences of these nurses? Would this warrant another study? Could this have been incorporated into the present study to yield richer and more comprehensive results if a broader sampling technique had been employed?

4 Collect data.

How will I gather the participants' descriptions of the event, experience or phenomenon being studied to answer the research question?

Data collection in qualitative descriptive studies may include semi‐structured, individual interviews, open‐ended questionnaires and focus groups (Colorafi and Evans 2016; Kim, Sefcik, and Bradway 2017; Sandelowski 2000). Interviews (individual or focus groups) are the most frequently used method as they enable researchers to probe and explore issues, encouraging depth of understanding and contributing to the richness of data (Birks, Hoare, and Mills 2019; Bradshaw, Atkinson, and Doody 2017). Researchers must justify the choice of conducting a focus group or an interview, as the inappropriate use of either may diminish the data quality (Cleary, Horsfall, and Hayter 2014). For example, focus groups may be beneficial when seeking collective perspectives, behaviours and attitudes through group dynamics, such as in the case of understanding issues faced by migrant nurses when caring for palliative aged care patients (Angus et al. 2021) or in the exploration of international nursing students' experiences of team learning (Randall, Crawford, and River 2020). However, focus groups may not allow full exploration of personally sensitive topics, such as in describing career challenges and hardships of internationally educated nurses (Miyata 2023) or in describing migrant care workers' perceptions of their job demands and their intentions to leave their current employment (Adebayo et al. 2023).

Data quality is also connected to the interviewer's skills and knowledge. The dialogue between the participant and researcher may determine the breadth and depth of the data gathered, wherein researchers who probe insufficiently may risk obtaining poor information or superficial data (Cleary, Horsfall, and Hayter 2014; Malterud, Siersma, and Guassora 2016; Morse 2015a). Novice researchers have reflected on the challenges of interviewing participants, resulting in failing to ‘dig deep’ (Wolfhart 2020, p.2) or failing to develop rapport and obtain sufficient information (Kalman 2019). Poor quality of data may result in study findings that are already known and, therefore, do not contribute to existing knowledge or add new knowledge (Roberts 2020), stressing the importance of interviewer training. Honing interview skills in the novice researcher is beyond the scope of this paper, but there are several guides for this (e.g., see Fusch et al. 2022; McGrath, Palmgren, and Liljedahl 2019; Roberts 2020; Whiting 2008).

In our current research, we opted to gather data using individual, semi‐structured interviews as some experiences and challenges may be too personal or deemed sensitive by participants and, thus, may be inappropriate to discuss openly in a group setting, such as in focus groups. While focus groups may provide collective perspectives, this may not enable us to explore and probe individual experiences and challenges, possibly diminishing data quality. To maintain consistency, the research team developed an interview guide informed by the literature and our chosen theoretical framework (SDT). Broadly, interview questions were about motivations for migration, experiences after arrival, differences and similarities between current practice and previous nursing practice, relationships and interaction with co‐workers, patients and managers, and socialisation and relationships within and outside the workplace. Pilot interviews (excluded from the interview count) were conducted to make sure that the interview questions were unambiguous.

5 Analyse data.

How will I analyse the participants' descriptions of the event, experience or phenomenon being studied to answer the research question?

Data analysis in qualitative descriptive design is often achieved through thematic or content analysis, as both methods offer the level of interpretation necessary in qualitative description (Doyle et al. 2020; Vaismoradi, Turunen, and Bondas 2013). Regardless of the analytic method, data analysis aims to identify participants' ideas and perspectives to form detailed, clear and thick descriptions of the event being studied (Sullivan‐Bolyai and Bova 2021).

Thematic analysis is an analytic method wherein a researcher identifies, analyses, describes and interprets patterns of meaning from the data (Braun and Clarke 2006, 2020). Content analysis (sometimes called qualitative content analysis) is a technique to analyse and categorise words in a data set, identifying their frequencies, relationships and patterns of occurrence (Colorafi and Evans 2016; Vaismoradi, Turunen, and Bondas 2013). This section delves into the concepts behind data analysis and how they are applied in qualitative descriptive studies (see the following resources for content or thematic analysis: Braun and Clarke 2006, 2020; Burnard 1991; Byrne 2022; Colorafi and Evans 2016; Elo and Kyngäs 2008; Vaismoradi, Turunen, and Bondas 2013).

Prior to data analysis, researchers should first decide whether the analysis focuses on latent or manifest content, using an inductive or deductive approach (Bengtsson 2016; Graneheim and Lundman 2004; Graneheim, Lindgren, and Lundman 2017; Vaismoradi, Turunen, and Bondas 2013). A data‐driven (inductive) approach is characterised by searching patterns in the data and may be utilised when research on the topic or extant theory is limited (Bengtsson 2016; Graneheim, Lindgren, and Lundman 2017; Hsieh and Shannon 2005). A concept‐driven (deductive) approach is characterised by using an existing theory, wherein researchers expand or test the existing theory against the collected data (Graneheim, Lindgren, and Lundman 2017; Hsieh and Shannon 2005). Since qualitative descriptive analysis allows flexibility around the use of theory, data analysis could use either inductive or deductive reasoning.

Manifest content refers to evident components of the text, wherein the usage, frequency of patterns and regularities confirm the description of events (Graneheim and Lundman 2004; Sandelowski 2000). In contrast, latent content moves beyond quantifying means and frequencies of responses to interpreting the underlying sense of the text (Graneheim and Lundman 2004; Hsieh and Shannon 2005; Kleinheksel et al. 2020). Content analysis may focus on latent or manifest content, while thematic analysis incorporates both, such that the latent content is inseparable from the manifest content of data (Vaismoradi, Turunen, and Bondas 2013). There are various approaches to content analysis (Burnard 1991; Elo and Kyngäs 2008; Graneheim and Lundman 2004; Hsieh and Shannon 2005) and thematic analysis (Braun and Clarke 2006; Coates, Jordan, and Clarke 2021). Regardless of which analytic approach is chosen, researchers should clearly state which method they employed to maintain research transparency and enhance rigour.

Another concept to clarify is the identification of findings as categories or themes. A category refers to a collection of similar opinions, attitudes and experiences, often referred to as the manifest content, while a theme is described as an essence running through several categories or the latent content (Graneheim, Lindgren, and Lundman 2017; Morse 2015b; Vaismoradi, Turunen, and Bondas 2013). In thematic analysis, a theme is not contingent on the frequency of occurrence but on whether it represents something relevant to the research question (Vaismoradi, Turunen, and Bondas 2013).

Several studies will be presented to illustrate data analysis in qualitative descriptive studies. In a study exploring the integration experiences of culturally and linguistically diverse (CALD) nurses in a foreign country, Kamau et al. (2023) utilised an inductive content analysis, deriving codes from data. The analytic process began by going through the transcribed text, after which the researchers derived 596 meaning units (collection of related words or statements), which were arranged into 359 codes (label of meaning units) (Kamau et al. 2023). Codes were analysed, categorised based on similarities and arranged according to the research question. This process narrowed their findings to 21 subcategories, which fell into eight categories (Kamau et al. 2023). Categories represent a collection of similar experiences or perceptions, such as discrimination, racism and cultural sensitivity being classified under the category ‘racial/ethnic experiences’ or career development, deskilling and workplace competence falling under the category of ‘professional competence development’ (Kamau et al. 2023).

Högstedt et al. (2021) also used inductive content analysis to describe the experiences of internationally educated nurses involved in a recertification process in a foreign country. As with the previous study, meaning units were identified, grouped and coded. The authors clearly described the process of coding similar content into categories to express the manifest content of their data, followed by grouping and linking categories (i.e., challenges in learning new skills and language and feelings of stress, anxiety and exhaustion) into subthemes (the constant struggle of an uncontrollable process) to identify a central theme (rollercoaster of emotions). This theme allowed them to express the latent content of their data.

Research by Zhong et al. (2023) described how migrant nurses evolved and adapted professionally and utilised thematic analysis (Braun and Clarke 2006, 2020) to interpret their data. The authors familiarised themselves with the data and used an inductive approach to identify their codes. The codes were collated, categorised into potential themes and reviewed against the entire data set to ensure that the identified themes represent the extracts. Modifications were made as necessary, leaving the researchers with three themes to summarise how migrant nurses adapted and evolved professionally (i.e., perceived differences, adaptation challenges and self‐evolution).

**FIGURE 1:**
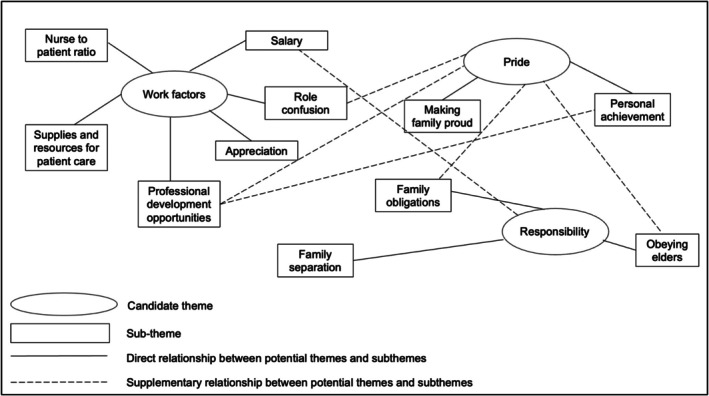
Initial thematic map. *Note:* This is not an exhaustive example and merely serves as a visual guide on how we identified the relationships between themes and subthemes and labelled potential themes.

In our current study, we used reflexive thematic analysis (Braun and Clarke 2006, 2022) in an inductive approach to develop codes from the participants' responses. Although an in‐depth explanation of thematic analysis is beyond the scope of this article (see earlier resources cited), we present a portion of our initial thematic map in Figure 1. Our final themes are presented in other papers (under review).

*Caveat: Be reflexive to avoid shaping results to fit familiar directions*.

Analysing large quantities of data and transforming them into findings are critical challenges in qualitative research (Cypress 2019; Magilvy and Thomas 2009). Coding frameworks and a priori rules may help sort and organise data; however, they may impede analytic creativity and prematurely disregard other ways of ‘seeing’ the data (Sandelowski 1995a). While frameworks enable data to be organised, the analysis must be data‐driven in any inductive research to maintain faithfulness to the data (Sandelowski 1993a, 1995a). Care also needs to be taken not to shape results to one's predispositions and biases, which have implications for the credibility of findings (Cypress 2019). The researcher is an instrument in a qualitative study, highlighting the importance of reflexivity, transparency of opinions and biases, and peer review to ensure findings are credible and valid (Cypress 2017; Willis et al. 2016).

6 Identify parameters for saturation (to determine sample size).
  When do I stop data collection?

Data collection and analysis occur simultaneously in most qualitative studies (Lambert and Lambert 2012). Ideally, data collection stops when further data will not provide additional depth to the study, producing redundant information (Cleary, Horsfall, and Hayter 2014; Moser and Korstjens 2018). According to Morse (1995), ‘an edict of qualitative research is to collect data until saturation occurs’ (p. 147). Saturation is achieved when all aspects and depths of the event have been explored and reported by multiple participants, and no new concepts materialise from succeeding interviews (Cleary, Horsfall, and Hayter 2014; Morse 2015a). Lack of saturation may result in researchers reporting findings from ‘thin’ data and yield invalid findings (Morse 1995, 2010). Researchers describing findings from saturated data no longer discuss individual cases but progress to explaining generalities (Morse 2015a).

Saturation is facilitated by an adequate and appropriate sample (Morse 2015a). An adequate sample size allows researchers to note replication in reports of participants wherein concepts are repeated multiple times, while an appropriate sample enables researchers to collect data from those who can provide first‐hand accounts of the event being explored (Cleary, Horsfall, and Hayter 2014; Morse 2015a). Saturation is also called information redundancy, where researchers can anticipate information from repeatedly hearing or seeing data, so more data collection does not add further value (Sandelowski 2008). Sandelowski (1995b) asserts that experience plays a role in determining whether saturation or information redundancy has been achieved. Hence, data collection does not stop when data have been gathered from the pre‐set sample size but rather when researchers are satisfied with the depth, adequacy and appropriateness of the data (Cleary, Horsfall, and Hayter 2014; Morse 2015a; Moser and Korstjens 2018).

Saturation is a crucial component of rigour in qualitative studies (Morse 1995); however, its use in qualitative research apart from grounded theory is considered by some as unsuitable, owing to ambiguity around the concept and the fact that qualitative research focuses on individuals' experiences, thereby saturation may never indeed be reached (Bradshaw, Atkinson, and Doody 2017; O'Reilly and Parker 2012). In an attempt to clarify the concept, scholars have coined the terms code saturation or inductive thematic saturation (further data does not lead to new codes), a priori thematic saturation (current data exemplifies pre‐identified codes or themes), meaning saturation (further data does not lead to new dimensions or insights), theoretical saturation (further data does not enhance dimensions of categories) and data saturation (new data repeats previously expressed data) (Hennink, Kaiser, and Marconi 2017; Saunders et al. 2018). On the other hand, Malterud, Siersma, and Guassora (2016) proposed ‘information power’ rather than saturation as a guide for researchers to identify when to stop sampling. These terms demonstrate the diversity in saturation parameters, although their commonalities lie where further data do not lead to new codes, meanings, themes, concepts or categories (Sebele‐Mpofu and Serpa 2020).

In qualitative descriptive studies, the terms thematic saturation or data saturation are frequently used wherein these are reported as having been achieved after no new themes (Almansour, Gobbi, and Prichard 2021; Randall, Crawford, and River 2020) or no new aspects (Zhong et al. 2023) emerged from interviews. Some studies mention achieving saturation but do not elaborate on how (Kiviniitty et al. 2023; Miyata 2023), while some studies do not mention saturation entirely (Högstedt et al. 2021; Kelleher, FitzGerald, and Hegarty 2016).

The parameters of saturation differ depending on the research design, theoretical or conceptual underpinning, and research methods (Saunders et al. 2018). As in any research, the goal is to obtain rich information to answer the research question, and the onus of researchers is to defend their sampling strategies, clearly articulate their notion of saturation, and define their parameter for achieving saturation (Bradshaw, Atkinson, and Doody 2017; Patton 2002; Sandelowski 2000; Saunders et al. 2018).

In our current research example, we anticipated a sample size of 15–30 participants, as previous studies identified that saturation from in‐depth interviews was attained between 9 and 17 interviews (Hennink and Kaiser 2022). Our final sample included 17 migrant nurses. During analysis, we developed 81 codes from the participants' responses and narrowed them down to 62 codes after repeated rounds of coding. By the 14th interview, we could code the entire transcript into the codes developed from earlier interviews. In the succeeding interviews, we found that we could not develop new codes, so we ceased data collection, closing our study with 17 participants.

*Caveat: A pre‐determined sample size may be required for a data proposal, but the actual sample size may differ*.

Determining a sample size in qualitative research has been debated (Bradshaw, Atkinson, and Doody 2017; Hennink and Kaiser 2022; O'Reilly and Parker 2012). Scholars have proposed that an adequate sample size provides rich, comprehensive, and information‐packed data relevant to the study, enabling the researchers to answer the research questions (Bradshaw, Atkinson, and Doody 2017; Malterud, Siersma, and Guassora 2016). A systematic review of qualitative descriptive studies identified a sample size range between 8 and 1932 participants, with the most common sample size being 11–20 (Kim, Sefcik, and Bradway 2017).

Strategies to collect data may also influence sample size (Morse 2015b). Studies with larger sample sizes are conducted using open‐ended survey questionnaires or focus groups (Kim, Sefcik, and Bradway 2017). The amount of information obtained from each participant in semi‐structured interviews is restricted compared to unstructured interviews and often requires the researcher to recruit more participants to obtain adequate data (Morse 2015a, 2015b). Focus groups may yield fragmented data (Morse 2015b); hence, researchers may need to conduct multiple focus groups to collect sufficient information. Pre‐determining a sample size range is often necessary for submitting a research proposal, and the abovementioned studies may provide good approximations. Nonetheless, rather than adhering to the pre‐set target, researchers should look at the quality of their data as a guide on when to stop their data collection.

7 Present the findings.

How will my writing reflect the participants' descriptions of the event, experience, or phenomenon being studied to answer the research question?

A qualitative descriptive design allows for researcher creativity when presenting data if the findings accurately reflect participants' voices, are presented as straightforward accounts of participants' experiences, and are organised logically (Colorafi and Evans 2016; Neergaard et al. 2009; Sandelowski 2000). Findings can be presented (summarised) as (1) a timeline of events, (2) major categories, (3) most predominant to least predominant themes, (4) a broad to narrow context of an event or specific case or vice versa, or (5) describing the event from perspectives of several participants (Lambert and Lambert 2012; Sandelowski 2000). Despite the numerous ways to organise findings, presenting the data in themes or categories seems to be the most frequent way of doing so (Kim, Sefcik, and Bradway 2017).

Sandelowski (2007) warns against the excessive use of analytic words (i.e., explaining how categories emerged from data or how codes collapsed into concepts), as these may obscure the meaning of data, conceal research findings, and do little to help convey participants' voices. In qualitative descriptive studies, the data are the star, and the researcher may take the stance of a reporter and interpreter (Sandelowski 1998). Ideally, data are not presented in any other terms apart from their own, whereby ‘concerns remained concerns and perceptions remained perceptions’ (Sandelowski 2000, p.338). For instance, as we explored the experiences of migrant nurses to understand overall factors that lead to workplace retention, we presented these experiences as (1) before migration (to understand motivations), (2) during relocation (to explore transition challenges), and (3) after transition (to understand experiences that lead to retention). Reflecting Sandelowski's terms, experiences remained experiences.

*Caveat: Participant quotations are used to strengthen findings, not to satisfy reporting checklists*.

Participant quotations in qualitative research are anticipated and expected (Eldh, Årestedt, and Berterö 2020; Sandelowski 1994). Traditionally, quotations provide a concrete example of participant experience or evoke emotion (Sandelowski 1994); however, recent literature calls for methodological justification in using quotations (Daly 2009; Eldh, Årestedt, and Berterö 2020; Thorne 2020). The Consolidated Criteria for Reporting Qualitative Research (COREQ) (Tong, Sainsbury, and Craig 2007), a commonly used checklist for judging qualitative research, assesses whether supporting quotations are provided and whether these are adequately identified. Per the checklist, quotations add transparency and trustworthiness to qualitative research findings and interpretation (Tong, Sainsbury, and Craig 2007). Although well‐meaning, this may drive researchers to include quotations ‘to meet the checklist’ rather than to justify and strengthen their findings. Instead of focusing on satisfying reporting criteria or meeting conventional expectations in qualitative research, researchers should identify the purpose of the included quotation—Does it represent a theme, category, or common view? Does it assist the reader in understanding the logic of analysis? Does it illustrate conflicting participant viewpoints (Daly 2009; Eldh, Årestedt, and Berterö 2020; Thorne 2020)? This way, quotations are used purposefully and give readers a better account of the studied event.

8 Maintain rigour throughout the study.

How will I ensure my research is of high quality (i.e., rigorous)?

Rigour is characterised by the strength and quality of the research design and suitability of the methods and is often seen as a marker of excellence, ensuring that the research process and outcomes are reliable, valid, and trustworthy (Cypress 2017; Morse 2015c; Smith and McGannon 2018). The ‘gold standard’ for establishing rigour in qualitative research includes demonstrating (1) credibility (internal validity), which refers to the truth in research interpretation, presentation of findings and value of data, (2) dependability (reliability), or the stability of data over various conditions, (3) confirmability (objectivity), which is the reflection of findings as the participants' and not the researchers', and (4) transferability (external validity), the extent to which study findings apply in other groups or settings (Lincoln and Guba 1985, 1986; Polit and Beck 2022).

Creswell (2012) suggests that researchers use at least two strategies in any study. Researchers should be mindful that although various techniques to achieve rigour have been identified, strategies should be congruent with the study design (Baillie 2015). Sandelowski (1993b) also cautions against inadvertently subduing the spirit of qualitative work by the uncritical application of rules to achieve rigour. Ultimately, researchers should conduct the study with integrity, approach every step of the research process thoughtfully, and document all efforts to achieve rigour (Baillie 2015; Johnson, Adkins, and Chauvin 2020). Careful deliberation and researcher reflexivity are crucial to establishing rigour (Morse 2015c). The strategies for achieving rigour are explained further in Table 1.

**TABLE 1:** Strategies to achieve rigour in qualitative descriptive studies.

Strategy	Description	Criteria	Comments
Prolonged engagement	Lengthy and intensive contact with participants to build trust and establish rapport	Credibility	During data collection, increased trust and established relationships may predispose participants to disclose more in‐depth descriptions, giving richer data.
Triangulation	Using two or more researchers to analyse and interpret data (investigator triangulation) OR using multiple data sources to validate data (data triangulation)	Credibility and Confirmability	Triangulation can be done in four ways: data, investigator, theory, and method (Denzin 1989). In qualitative descriptive studies, method triangulation would not be applicable unless conducting a mixed‐methods study. Similarly, theory triangulation may not apply as it may stifle the data and prevent researchers from staying close to participants' descriptions of events.
Peer review or debriefing	Presenting findings to a neutral peer or conducting regular meeting sessions with co‐researchers and supervision team (post‐graduate study)	Credibility	Peer review allows an external check on the research process, challenges the researchers, and mitigates researcher bias.
Negative case analysis	Actively seeking data that does not fit the researcher's interpretation	Credibility	Attention to data that does not fit the emerging pattern enriches understanding of the event as a whole.
Member check	Allowing participants to check their interview responses, either through playing back interview recording, giving transcribed interviews or completed analysis	Credibility	There are concerns that participants may be unable to recognise their own stories or remember their interview responses. Returning responses to participants also imposes on participants' time. An alternative is to probe and repeat participant responses during interviews to ensure adequate understanding and verify data accuracy.
Thick description	Sufficient details of participants, setting, and context in which the study occurs, including field notes	Transferability	Thick description allows readers to recreate the study and/or judge on whether findings are transferrable.
Audit trail	A record of all stages of the research, including notes, decision guides, raw data, drafts of data analysis, and changes made throughout the study	Dependability Confirmability	Provides a methodological description of processes and decision‐making throughout the study, identifying the rationale behind decisions and the researcher's bias, reflections, and insights. Allows others to follow the decision trail and arrive at the same results.
Reflexivity	Critical reflection of oneself as a researcher, including identifying bias, values, preconceptions or behaviour	Credibility Dependability Confirmability Transferability	Reflexivity through the use of reflexive journals or memoing is central to audit trails and supports the researcher to manage oneself as a ‘human instrument’, and to actively reflect on their biases and how these influence the research process and affects the study's rigour.

*Note:* Table developed by the authors using scholarly works from Baillie (2015), Bradshaw, Atkinson, and Doody (2017), Colorafi and Evans (2016), Johnson, Adkins, and Chauvin (2020), Lincoln and Guba (1985, 1986), Milne and Oberle (2005), Morse (2015c), Nowell et al. (2017), and Polit and Beck (2022).

Qualitative descriptive studies have also utilised several other techniques to achieve rigour. These include presenting direct quotations from participants in study findings to enhance confirmability and credibility (Almansour, Gobbi, and Prichard 2021; Bradshaw, Atkinson, and Doody 2017; Javanmard et al. 2020; Milne and Oberle 2005) and using the same questionnaire or interview guide to demonstrate consistent data collection and maintain dependability (Colorafi and Evans 2016). Strategies to achieve rigour should be explained in the data analysis section or before the presentation of findings.

*Caveat: While the established “gold” standard for determining rigour is Lincoln and Guba's criteria, some scholars call for a return to validity and reliability*.

Historical debates on determining rigour in qualitative research led Lincoln and Guba (1985) to develop a counterpart for quantitative research's criteria of validity and reliability (Morse et al. 2002), the accepted ‘gold standard’, as previously discussed. However, the applicability of validity and reliability in qualitative research is being re‐examined (Cypress 2017; Morse 2015c, 2017). Despite varying terminologies, some strategies to achieve rigour are similar, such as thick description, prolonged engagement, triangulation, negative case analysis and peer review. As such, researchers should review relevant literature to identify which view they align with and remember that the goal is to enhance rigour regardless of the differences.

### 4.3 Process of Conducting a Qualitative Descriptive Study

**FIGURE 2:**
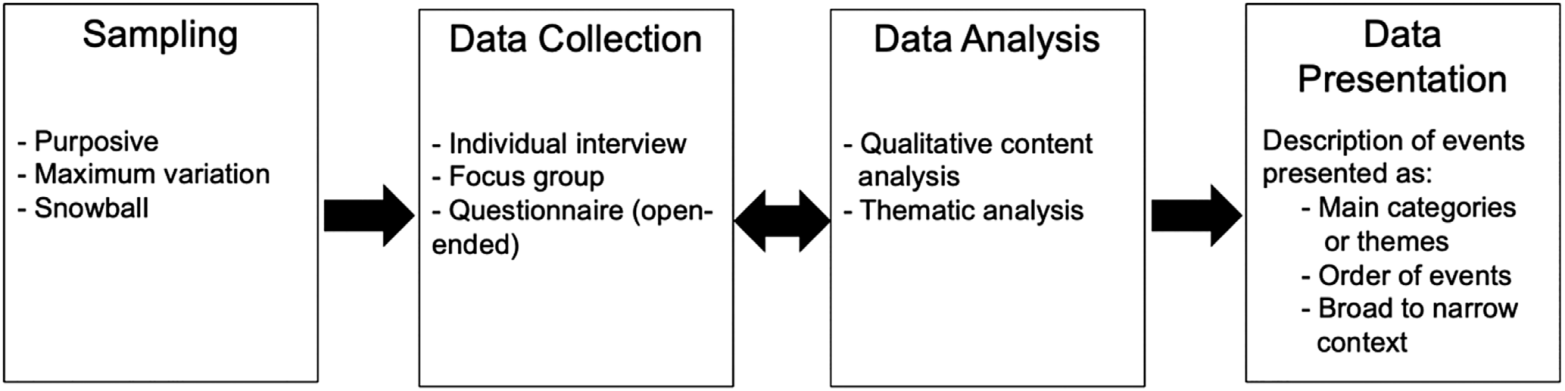
Qualitative descriptive study: Summary of options for conducting a qualitative descriptive study. *Note:* The figure summarises options for methods used in conducting a qualitative descriptive study. The two‐sided arrow between data collection and analysis signifies that these steps co‐occur. The figure is informed by the works of Bradshaw, Atkinson, and Doody (2017), Colorafi and Evans (2016), Doyle et al. (2020) and Sandelowski (2000).

**FIGURE 3:**
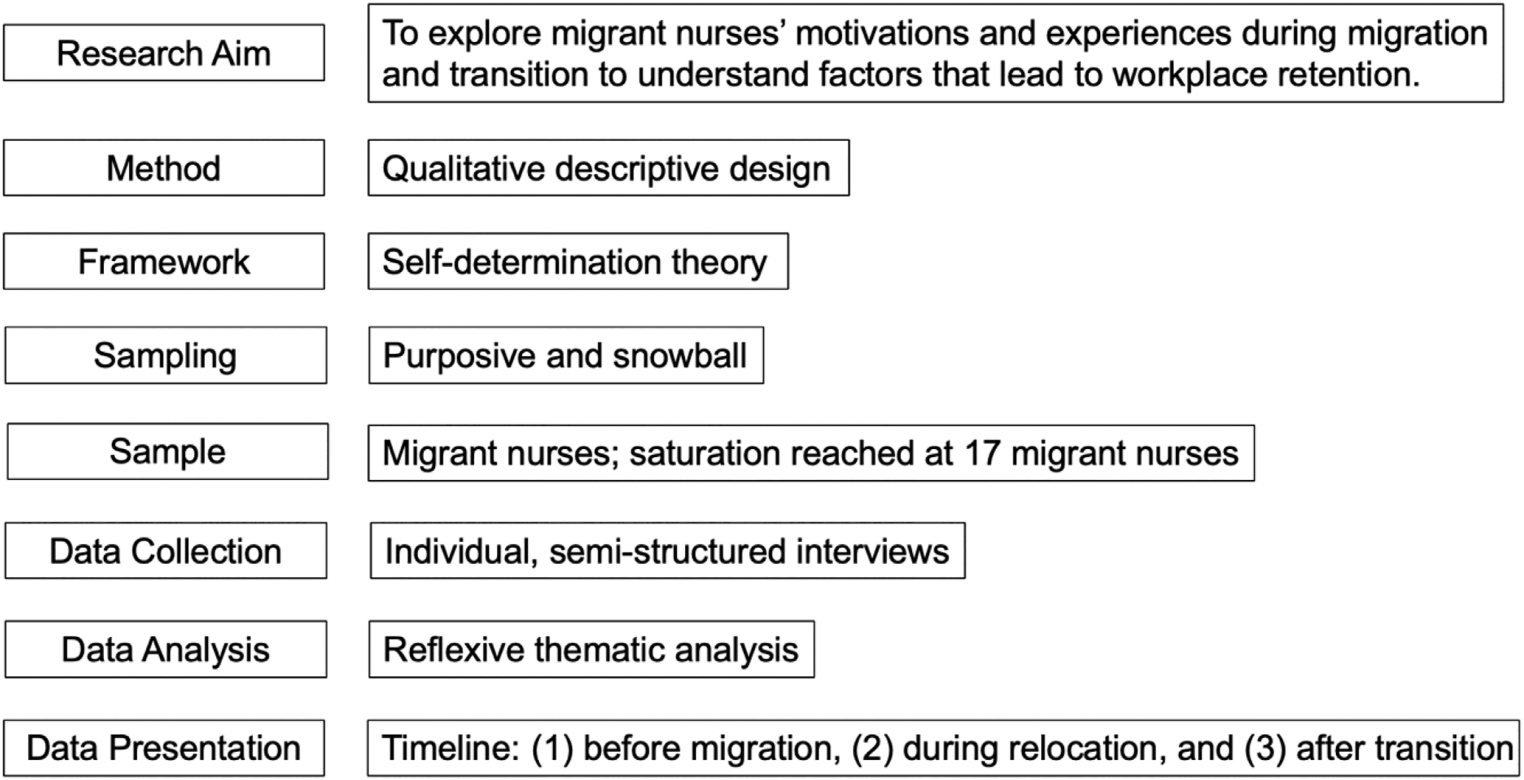
Qualitative descriptive study: Summary of the worked example.

Figure 2 summarises the options for conducting a qualitative descriptive study, while Figure 3 provides a summary of the worked example.

## 5 Conclusion

Qualitative descriptive design is widely used in nursing and health science research. The flexibility of the design may often cause confusion, which may lead to a poorly conducted study that does not give justice to qualitative descriptive design nor contribute to knowledge formation. Clarifying the steps involved may aid novice and early career researchers when conducting qualitative descriptive studies thoughtfully and with methodological rigour.

Without a proper understanding of the nuances of qualitative descriptive study, novice and early career researchers may be caught in some of the pitfalls and perils of this method. This includes the risk of using rigid sampling techniques, terminating data collection without reaching saturation, prematurely completing the analysis and presenting data that does not stay true to participants' descriptions. These may limit the scope of research and, subsequently, the quality and impact of the findings.

Qualitative descriptive design often uses purposive, snowball or maximum variation sampling. This design allows flexibility around the use of theoretical frameworks in that the researcher may abandon or revise the framework if, at any point, the research leads elsewhere and no longer aligns with the framework. Data collection is commonly done through focus groups or semi‐structured interviews and analysed using content or thematic analysis. Findings are organised to answer the research question and presented as close to the participants' descriptions as possible. Rigour throughout the research ensures that results are credible, dependable, confirmable and transferrable. Some strategies to ensure rigour include providing ample details to allow research replication, establishing an audit trail to include decisions made throughout the study and ensuring researcher reflexivity.

Conducted with rigour and research integrity, qualitative descriptive design is a valuable method, providing first‐hand descriptions of events, experiences or phenomena being studied. These accounts enable researchers to gain a straightforward and comprehensive understanding, which, in turn, may yield impactful findings that may contribute to change and/or new knowledge or serve as a foundation for larger‐scale studies.

## Author Contributions

Made substantial contributions to conception and design, or acquisition of data or analysis and interpretation of data: P.V., M.C., V.L., D.K.T. Involved in drafting the manuscript or revising it critically for important intellectual content: P.V., M.C., V.L., D.K.T. All authors have approved the final version to be published.

## Conflicts of Interest

The authors declare no conflicts of interest.

## Data Availability

The authors have nothing to report.
